# In Situ Smoothing of Facets on Spalled GaAs(100) Substrates during OMVPE Growth of III–V Epilayers, Solar Cells, and Other Devices: The Impact of Surface Impurities/Dopants

**DOI:** 10.1021/acs.cgd.3c01407

**Published:** 2024-04-01

**Authors:** William E. McMahon, Anna K. Braun, Allison N. Perna, Pablo G. Coll, Kevin L. Schulte, Jacob T. Boyer, Anica N. Neumann, John F. Geisz, Emily L. Warren, Aaron J. Ptak, Arno P. Merkle, Mariana I. Bertoni, Corinne E. Packard, Myles A. Steiner

**Affiliations:** † National Renewable Energy Laboratory, Golden, Colorado 80401, United States; ‡ Colorado School of Mines, Golden, Colorado 80401, United States; § Crystal Sonic Inc., Phoenix, Arizona 85003, United States; ∥ Arizona State University, Tempe, Arizona 85287, United States

## Abstract

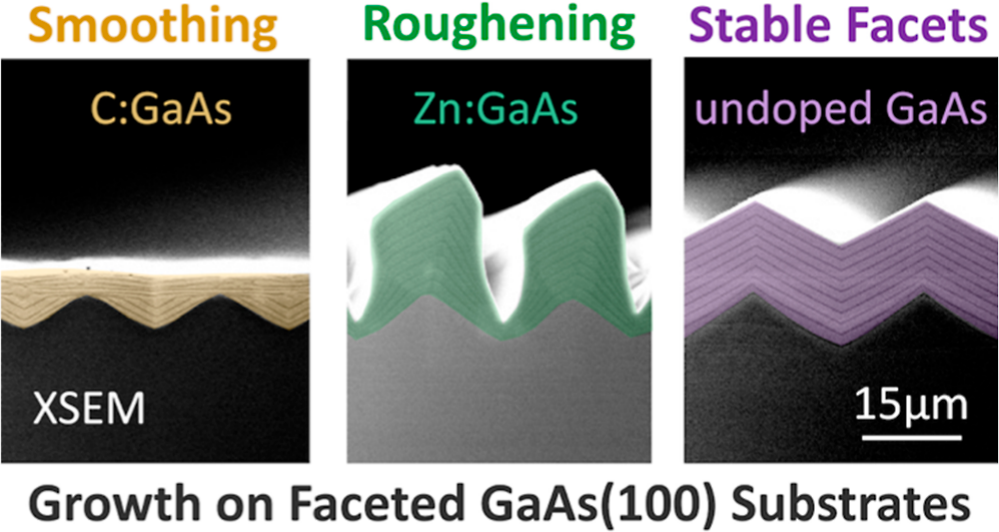

One possible pathway toward reducing the cost of III–V solar cells is to remove them from their growth substrate by spalling fracture, and then reuse the substrate for the growth of multiple cells. Here we consider the growth of III–V cells on spalled GaAs(100) substrates, which typically have faceted surfaces after spalling. To facilitate the growth of high-quality cells, these faceted surfaces should be smoothed prior to cell growth. In this study, we show that these surfaces can be smoothed during organometallic vapor-phase epitaxy growth, but the choice of epilayer material and modification of the various surfaces by impurities/dopants greatly impacts whether or not the surface becomes smooth, and how rapidly the smoothing occurs. Representative examples are presented along with a discussion of the underlying growth processes. Although this work was motivated by solar cell growth, the methods are generally applicable to the growth of any III–V device on a nonplanar substrate.

## Abstract 2

Although most epitaxial growth is done on planar surfaces, there are situations where growth on a nonplanar surface is necessary or desirable. In this study, we show how faceted surfaces can be smoothed during organometallic vapor-phase epitaxy growth and how the choice of epilayer material and modification of the various surfaces by impurities/dopants greatly impacts the evolution of the surface morphology.

## Introduction

Although most epitaxial growth is done on planar surfaces, there are situations where growth on a nonplanar surface is necessary or desirable. The research presented in this paper is directed toward reducing the cost of III–V solar cells by enabling the reuse of GaAs substrates, which (if not reused) comprise approximately one-third of the total cost of each final solar cell.^1^ However, the results and methods should be extensible to other III–V devices grown on nonplanar substrates.

**Figure 1:**
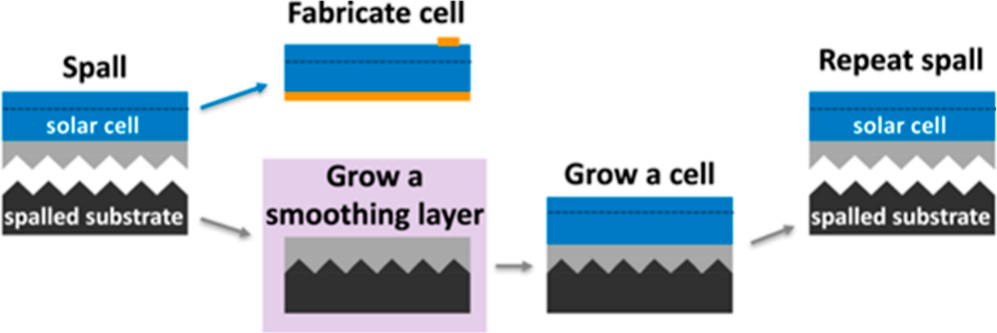
Schematics showing the basic processing steps needed to enable substrate reuse via spalling. For GaAs(100) substrates, the spalling process creates a faceted surface that must be smoothed before growing the next device. This work investigates how spalled GaAs(100) surfaces can be smoothed using OMVPE-grown epilayers.

Spalling provides an economical method for removing III–V solar cells from substrates such that each substrate can be reused multiple times, as shown in Figure 1.^2^ Ideally, the spall would travel parallel to the surface to create a flat planar surface to facilitate the growth of the next cell. However, the surface of a GaAs(100) substrate after spalling is not intrinsically flat, because (100) is not a natural cleavage plane for GaAs, and as such, achieving flat surfaces would require ultimate control of crack dynamics.^3^ For cases in which spalling does not produce a flat surface, the spalled surface tends to consist of a periodic array of faceted ridges, with facet directions lying on or near natural GaAs cleavage directions.^4^ For example, when a GaAs(100) substrate is spalled with a spall front traveling along a ⟨011⟩ direction, the result is typically a corrugated surface exposing {211} facets/planes.^5^

In this article, we demonstrate how surface smoothing during organometallic vapor phase epitaxy (OMVPE) growth is affected by two principal factors: (1) the crystal orientation of the surface facets and (2) the dopant/material combination used for the smoothing epilayers. The connection between these two factors and the underlying surface smoothing processes is also discussed at a phenomenological level. Examples will be presented to illustrate the wide range of outcomes that can be obtained by making different choices for the above two factors. Results for solar cells grown on spalled GaAs(100) substrates using the principles illustrated here have been presented elsewhere,^6^ and their performance is comparable to baseline devices grown on standard epiready GaAs(100) substrates.

## Experimental Details

### Sample Preparation

Two different spalling methods were used to prepare GaAs(100) substrates for subsequent growth studies. “Controlled spalling” uses a stressed-Ni layer and a roller to peel off a layer in a well-defined direction.^7^ “Acoustic spalling” uses a different stressor material, then applies “packets” of stress acoustically to control the velocity of the crack front as the surface layer is spalled off.^8^ In this paper, all samples were made using a controlled spall, except for those shown in Figure 10. The basic results presented here are agnostic to the process generating the surface morphology and should therefore be applicable to any spalling method.

All results in this study are for an as-spalled material with no additional surface preparation/cleaning, to ensure that the starting surface was similar for all cases. Also, we chose to use samples with ridges that were ∼5 μm high so as to be able to clearly observe morphology changes with cross-sectional scanning electron microscopy (XSEM). However, it is important to note that this does not represent the smoothness limits attainable by spalling methods.^7^ For actual cell growth, a pregrowth smoothing etch has been shown to be beneficial; this was investigated in a related study.^9^

This study evaluates GaAs, Al_0.43_Ga_0.57_As (hereafter “AlGaAs”), and GaInP_2_ (hereafter “GaInP”) as possible smoothing materials, doped with the various dopants being tested for their efficacy at smoothing the surface.

To study these morphology changes, thin (0.2 μm) marker layers were included periodically in each growth. One marker layer was grown after each 1 μm of the test material being studied; this was repeated 10 times so that each sample contained 10 marker layers. [These layer thicknesses are the nominal thicknesses which would be observed on a standard planar (100) substrate. As will be seen, the growth rates at different locations on a faceted substrate can be quite different due to lateral adatom diffusion and differences between sticking coefficients on different facets.] For most samples, the marker layers were AlGaAs, but GaAs marker layers were used for samples with AlGaAs test layers to provide contrast during XSEM imaging. In all cases, the dopant used in the test layer was also used in the marker layers to maintain a steady supply of dopant atoms to the surface.

III–V epilayers and cells were grown in an atmospheric-pressure OMVPE chamber at 650 °C, using trimethylgallium (TMGa), triethylgallium (TEGa), trimethylaluminum, trimethylindium, AsH_3_, PH_3_, CCl_4_, Si_2_H_6_, H_2_Se, and diethylzinc as sources. Except for the sample shown in Figure 10, the nominal growth conditions were as follows: GaAs {6 μm/h, V/III = 17}, AlGaAs {4 μm/h, V/III = 80}, GaInP {6 μm/h, V/III = 90}. TMGa was used for all layers except Zn:GaAs, which happened to use TEGa. The sample shown in Figure 10 was grown as part of a related study which used slightly different growth conditions (listed in the figure caption). Prior to growth, the samples were heated to 700 °C under an arsine overpressure and then held at 700 °C under arsine for 10 min to deoxidize the surface.

The doping levels used in this study were within the ranges generally used for standard device layers; no modifications to our OMVPE apparatus were made for this study. To facilitate replication of our results, we have provided nominal doping levels, defined to be the bulk doping concentration expected when using the same conditions to dope planar (100) epilayers with small offcut angles (i.e., less than 6°). This doping information is provided in the associated figure caption for each sample.

However, the bulk doping level simply provides information about the conditions used to grow each sample. As will be explained, what fundamentally matters is the alteration of the atomic structure of the various surfaces by dopant atoms at the surface. Because the associated surface reconstructions generally adopt specific elemental stoichiometries which are stable over a range of exposure conditions, the morphology changes observed in this paper should not depend sensitively upon the exact doping levels. It should also be mentioned that for any given growth/doping conditions, some variation in bulk dopant incorporation is expected as the crystallographic (faceting) direction of the surface is varied. This should not matter when the smoothing layers simply serve as buffer layers between an underlying spalled substrate and an overlying device. Applications for which the smoothing layers are also active optoelectronic device layers might require more careful calibration of the doping levels.

After growth, the samples were cleaved for XSEM imaging. In all samples, 0.1 μm of GaAs was grown as a buffer to bury any surface contamination before the first marker layer. In most cases, this slightly altered the surface morphology prior to growth of the first marker layer seen in the XSEM images. Electron channeling contrast imaging (ECCI) was also done to look for threading dislocations in the final epilayer.

### Spall Direction

**Figure 2:**
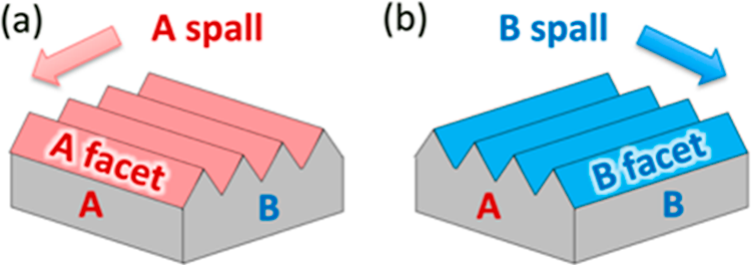
Diagrams show two principal spalling directions. For these two cases, the spalling direction determines whether any resulting facets are “Ga-terminated” {*n*11}A or “As-terminated” {*n*11}B facets.

When a GaAs(100) substrate is spalled, the (localized) direction of spall propagation determines the orientation of any resulting faceted ridges. Here we consider two possibilities. Figure 2a illustrates an “A spall”, which propagates toward an “*A*” direction, creating an array of ridges with {*n*11}A-faceted sides. Figure 2b shows a “B spall”, which propagates toward a “*B*” direction, creating an array of ridges with {*n*11}B-faceted sides.^5^ In both cases, there is no plane parallel to the original surface available for cleavage, so the propagating crack instead uses a set of low-energy planes near the depth dictated by the stress field applied during spalling.

Although the actual structure of a surface depends upon many factors, the “*A*” and “*B*” directions are qualitatively different and are often described, respectively, as “Ga-terminated” and “As-terminated” to indicate the difference in crystal polarity for the two directions. In our work, we found that the efficacy of a smoothing method could be quite different for different spall directions, presumably related to differences in the surface structure/composition of A versus B facets. The structural asymmetries between A and B surfaces can become even more dramatic once surface impurities/dopants are introduced, as they will generally incorporate into (and thereby change) the atomic structures of A and B surfaces differently. Other spall directions are possible but outside the scope of this study. For example, GaAs(100) spalled in an *AB* direction (midway between the *A* and *B* directions) will typically be faceted with {110} facets.^5^

## Results and Discussion

### Underlying Processes

**Figure 3:**
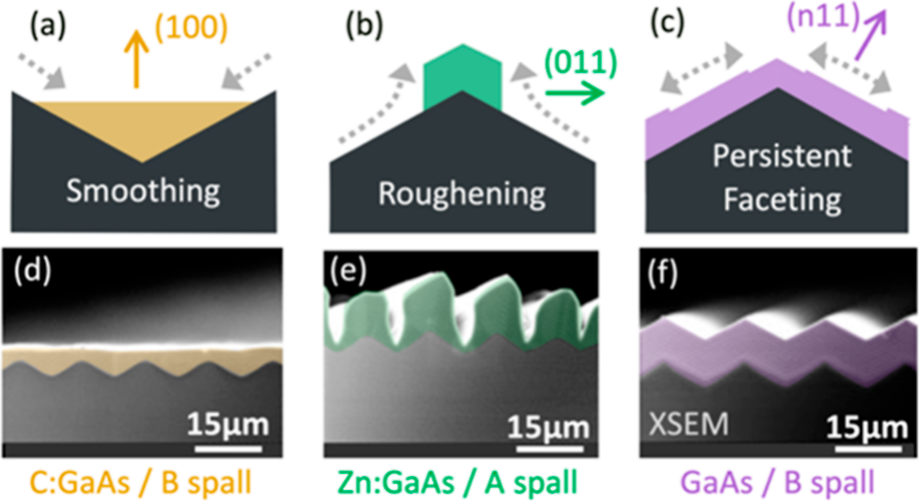
Schematics for three growth scenarios: (a) smoothing, (b) roughening, and (c) persistent faceting. The dashed gray arrows indicate how diffusion of surface adatoms can contribute to each growth mode. The stability of the facets labeled in each diagram can also contribute to each growth mode. (d–f) are XSEM images of samples exhibiting each growth mode for growth on a spalled GaAs(100) surface. Substrate offcuts: 6°A, 6°B, and 6°A for (d–f), respectively. The nominal doping concentrations for (d,e) were >10^19^ and mid-10^18^ cm^–3^, respectively; (f) was undoped. (Layers of interest have been colored for emphasis in this and subsequent figures.) Sample IDs: [MU368, 448, 370].

**Figure 4:**
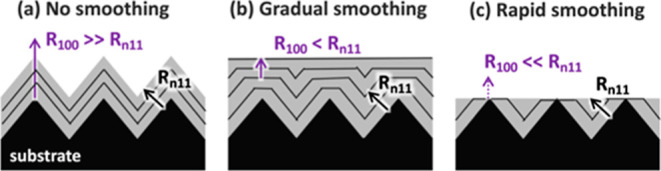
Schematics showing how the smoothing rate is affected by the relative growth rate of competing facets, where R_100_ and R_*n*11_ indicate the growth rate of the (100) and {*n*11} facets, respectively. The lengths of the arrows indicate the various growth rates. In (c), the growth rate for (100) facets is near zero. (See text for details.).

To facilitate the understanding of our experimental results, it is helpful to first consider the underlying processes. Changes in surface morphology during epilayer growth are driven by a combination of kinetic factors (affecting lateral surface diffusion of adatoms) and energetic factors (affecting sticking coefficients on different facets). Both factors can change the relative growth rate on competing facets, which, in turn, affects the morphological evolution of the surface. Figure 3 illustrates the connection between these underlying factors and three very different surface morphologies, with the focus primarily upon lateral surface diffusion of adatoms. Figure 4 provides a more empirical perspective that also encompasses inherent differences in facet growth rates due to differing sticking coefficients.

Figure 3a illustrates how a surface can be smoothed if the material diffusing across the surface primarily flows toward the bottoms of the trenches. This type of surface diffusion anisotropy can be caused by “Schwoebel” step energy barriers, which can allow diffusing surface adatoms to preferentially cross steps in one direction but not the other.^10−12^ Surface energetics can also drive this valley-filling growth mode. If the (100) surface is more stable than nearby surface facets, then it could grow more slowly such that the surface evolves to be entirely (100).

In Figure 3b, the surface diffusion anisotropy is reversed to allow mass transport toward the tops of the ridges, primarily causing surface roughening. This type of roughening can also be driven by surface energetics if steep side-facing facets [such as (011)] are stabilized (and thereby slow-growing).

Figure 3c illustrates a scenario in which {*n*11} side-facing facets [such as (211)] are very stable such that adatoms tend not to form new islands in the middle of a facet. Instead, the material diffuses to the edges of any incomplete facet, widening the facets until the surface consists of a periodic array of nearly perfect {*n*11} facets. Energetically, the stability of the {*n*11} facets also causes them to grow outward (as opposed to laterally) more slowly than other facets, such that they eventually become the dominant and persistent facet direction on the surface.

In all three scenarios, the underlying mechanisms (surface diffusion and facet stability) will be altered by changing the epilayer material, surface impurities/dopants, and/or facet orientation (e.g., A or B). Some representative examples are shown in the bottom row of Figure 3. Figure 3d shows how C:GaAs can flatten a B-spalled surface, Figure 3e shows Zn:GaAs growth roughening a B-spalled surface, and Figure 3f shows persistent faceting on an A-spalled surface for undoped GaAs. With regard to dopants, what matters most is that dopant atoms occupy surface sites as they are incorporated into the epilayer, and these surface-bonded dopant atoms can dramatically change the kinetic and energetic characteristics of the various surface facets. The bulk doping density estimates given for each sample simply provide information about the requisite growth conditions for each sample. Although we have no direct measure of the surface structure or composition, surface reconstructions driven by surface impurities typically contain a fraction of a monolayer of the impurity atoms, which is a much higher atomic fraction than a typical bulk doping density.

As seen in Figure 3, changes in the lateral adatom surface diffusion characteristics can dramatically change the way in which the surface morphology evolves. In many cases, it appears that the length scale for lateral mass transport is at least several microns. However, differences in sticking coefficient also affect the local growth rate on different facets. As a practical matter, it can be difficult or impossible to deconvolve the various factors, so it can also be helpful to view the problem in terms of the relative growth rates on competing facets.

This is shown in Figure 4, which illustrates how the rate of smoothing can be related to the growth rate of competing surface facets (where the growth rate is considered to be perpendicular to the plane of each facet). In all cases, the growth rate of any exposed (100) facet “R_100_″ is compared to the growth rate of any exposed {*n*11} facet “R_*n*11_″. These growth rates are observed/effective growth rates, including any changes in the growth rate due to lateral surface diffusion to/from each facet.

In Figure 4a, R_100_ ≫ R_*n*11_, such that if there were any exposed (100) facets, they would quickly grow upward until they vanished (as the adjacent {*n*11} facets become wider and converge to a pointed ridgetop). This situation could be caused by net surface diffusion from {*n*11} to (100) facets and/or a bigger sticking coefficient on the (100) facet.

In Figure 4b, R_100_ has been reduced to be slightly less than R_*n*11_. This impedes upward growth of (100) facets, causing them to become wider as growth proceeds. (At the atomic scale, the crystal lattice is discrete such that there is effectively always a small (100) facet atop the ridges, even if they look perfectly sharp in a simple schematic.) This situation could occur if the sticking coefficients for the two facets were similar and net surface diffusion between the facets were negligible. The result is nearly conformal growth, which gradually smooths the surface.

In Figure 4c, R_100_ was reduced to zero. This is a limiting case which could only happen if all of the adatoms landing on a (100) facet either diffused to an {*n*11} facet or returned to the vapor phase [due to a very low sticking coefficient on (100) facets]. In this case, the upward-facing (100) facets simply grow wider as the trenches are filled by growth on the {*n*11} facets. Once the trenches are filled, the (100) surface will cover the entire surface, such that diffusion from (100) to {*n*11} is no longer possible. After reaching this state (shown in Figure 4c), the (100) surface would begin to grow upward with a growth rate determined by the (100) sticking coefficient.

Finally, the use of ternary alloys can introduce additional complexity, in that lateral stoichiometric variation becomes a possibility. For example, prior work investigating AlGaAs growth on v-grooved substrates has observed and explained stoichiometric variations in the AlGaAs due to nonuniform lateral diffusion and incorporation of Ga and Al on various facets.^13,14^ Although it is inherently difficult to deconvolve stoichiometric nonuniformities from other effects, our principal results do not appear to be the result of stoichiometric nonuniformities. All structures in this paper include AlGaAs layers (either as marker layers or as test layers), yet a wide range of behavior is observed for the various dopant/material combinations being tested. Also, when the methods were applied to the growth of solar cells,^6^ AlGaAs marker layers were omitted and similar behavior was observed for the component epilayers. The quality of the resulting solar cells also indicates that any phase separation of the underlying GaInP smoothing layer was not enough to produce cell-degrading threading dislocations. Nonetheless, stoichiometric variation of ternaries can be a source of additional complexity, and this could favor the use of a binary compound such as GaAs as a smoothing layer.

### Undoped GaAs and GaInP

**Figure 5:**
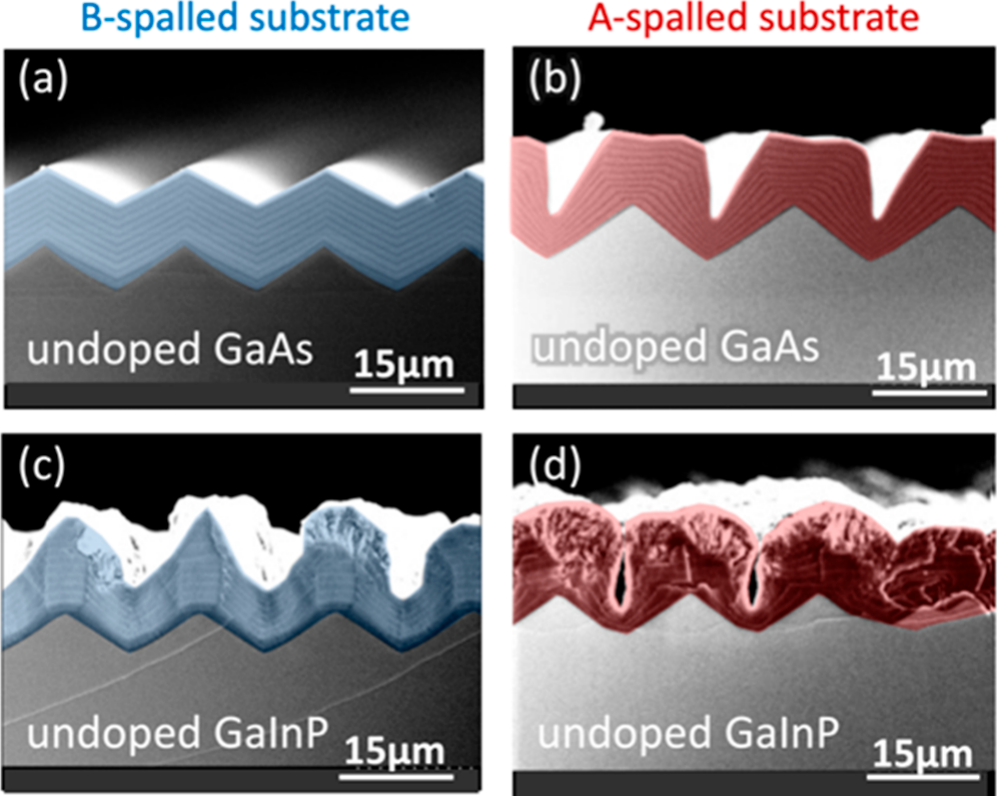
XSEM images for the growth of undoped GaAs and GaInP on spalled GaAs(100) substrates. Persistent faceting is seen in (a), and roughening is seen in (b–d). In this and all other XSEM images, thin (0.2 μm) GaAs marker layers indicate how the surface morphology evolved during the growth. Substrate offcuts: 6°A, 6°B, 6°A, and 6°B for (a–d), respectively. Sample IDs: [MU370, 446, 382, 452].

Figure 5 shows results for GaAs and GaInP grown on A- and B-spalled substrates with no intentional doping. For clarity, in this and subsequent figures, the epilayers above the first marker layer have been false-colored blue for B-spalled substrates and red for A-spalled substrates.

The growth of undoped GaAs on a B-spalled substrate (Figure 5a) creates very stable facets, which then persist. The final marker layers are very straight and parallel and do not simply replicate the original surface morphology. (The first marker layers are curved, and the final marker layers are straight.) Therefore, this faceting is due to the processes shown in Figures 3c and 4a, and it is not merely the result of conformal growth. Undoped GaAs grown on an A-spalled substrate (Figure 5b) roughens to have flat, nominally (100) ridgetops separated by deep trenches, suggestive of an anisotropic surface diffusion mechanism such as that shown in Figure 3b.

Undoped GaInP grown on a B-spalled substrate (Figure 5c) also seems to exhibit roughening from anisotropic diffusion but with the addition of “mushroom” formations atop the ridgetops. When undoped GaInP is grown on an A-spalled substrate (Figure 5d), this mushrooming worsens and the intervening valleys steepen into deep narrow trenches. It is interesting to note that trenches do not seem to form above shallower valleys, suggesting that better results might be obtained by (for example) using some sort of pregrowth smoothing etch.

None of the undoped surfaces in Figure 5 are flat or smooth, motivating subsequent experiments using doped materials.

### Si:GaAs and C:GaAs

**Figure 6:**
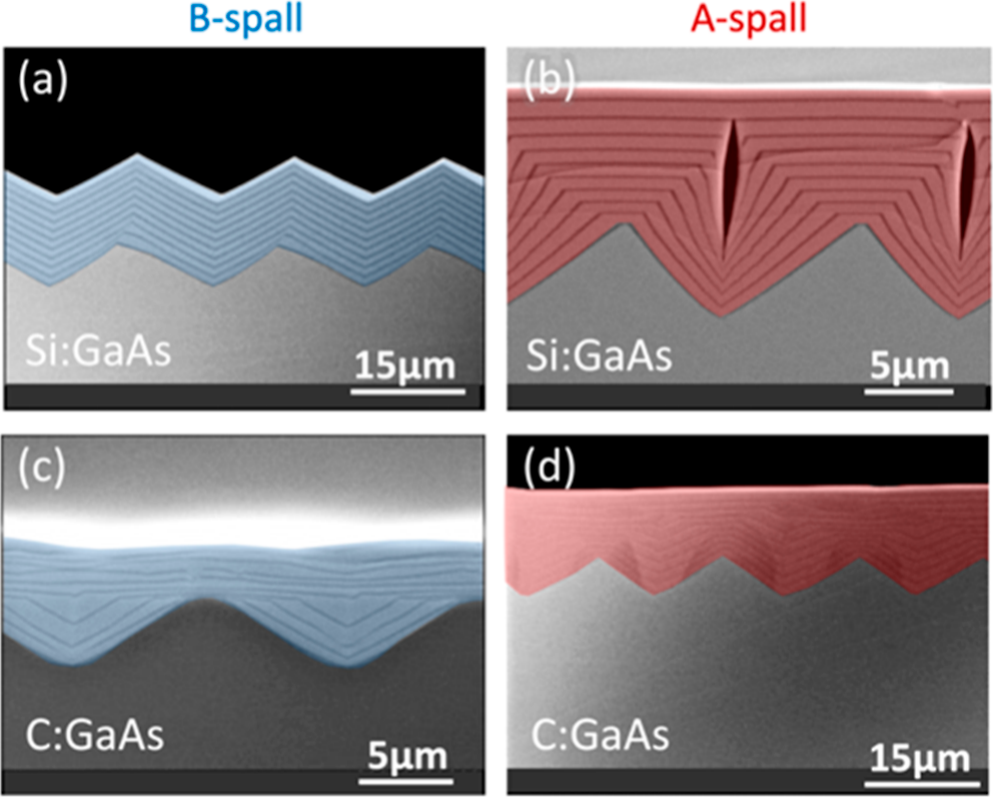
XSEM images for the growth of Si:GaAs and C:GaAs on spalled GaAs(100) substrates. Persistent faceting is seen in (a), and smoothing is seen in (b–d). The voids in (b) appear to be associated with substrate ridge heights greater than 5 μm. Substrate offcuts: 6°A, 0°, 6°A, and 6°B for (a–d), respectively. The nominal doping concentrations for (a–d) were approximately low-10^17^, high-10^17^, >10^19^, and >10^19^ cm^–3^, respectively. Sample IDs: [MU386, 734, 368, 444].

In Figure 6, the GaAs epilayers have been doped with Si and C, and these dopants clearly affect the morphological evolution. The use of Si and C dopants was inspired by prior studies in which group-IV exposure passivated and/or stabilized III–V(100) surfaces (Figure 5 in ref (15)), suggesting that they might promote the (100)-stabilized smoothing mechanisms shown in Figures 3a and 4c. Prior research has also shown that C:GaAs can be used to fill trenches patterned into GaAs(100) substrates.^16^ Our results show that both Si:GaAs and C:GaAs offer pathways toward spalled-substrate smoothing, with some different possible complications for each.

In Figure 6a, the Si:GaAs results appear identical to the undoped GaAs results in Figure 5a, suggesting that Si is not incorporating into the surface reconstruction of B facets or that the B facets continue to be very stable even with some Si at the surface.

The Si:GaAs grown on an A-spalled substrate (Figure 6b) does eventually flatten the surface but with some complications. It initially has deep trenches similar to the undoped GaAs in Figure 5b but with much flatter (100) ridgetops. However, the Si:GaAs trenches steepen more quickly and then close to form voids overgrown by a flat (100) surface. It seems likely that the void formation is related to some combination of anisotropic diffusion and a low surface energy for a near-vertical {011} facet, but the details of this are not yet understood.

Preliminary ECCI results showed no TD formation above the voids, so it might be possible to utilize these voids for some sort of device-related purpose (like light diffraction/scatter) or as a perforated weak layer for device liftoff. If the voids are deemed undesirable, they could be avoided by starting with a surface with shorter peak-to-valley heights. (An example of this will be shown later.)

The C:GaAs samples exhibit smoothing without void formation. Although the smoothing for C:GaAs/B-spall (Figure 6c) appears more rapid than for C:GaAs/A-spall (Figure 6d), both samples were supplied with the same nominal amount of material. It therefore appears that the sticking coefficient for Ga adatoms is lower for the C:GaAs/B-spall. The C:GaAs/B-spall sample also appears to be rougher. These differences might be partially related to the difference in substrate offcut direction, which was 6° toward (111)A for the B-spalled substrate in Figure 6c and 6° toward (111)B for the A-spalled substrate in Figure 6d.

### Zn:AlGaAs

**Figure 7:**
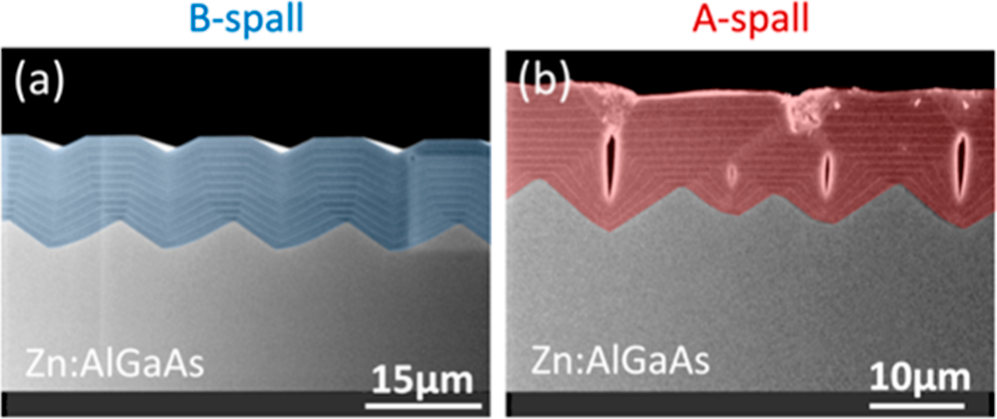
XSEM images of Zn:AlGaAs smoothing layers on spalled GaAs(100) substrates. Gradual smoothing is observed in both (a,b) but with the additional complication of void formation in (b). In (b) and other images of the same sample (not shown), the voids are smaller above shallower ridges, suggesting that void formation would be precluded if the original pregrowth surface were smoother, perhaps by changing the spalling conditions or by using a pregrowth smoothing etch. Substrate offcuts: 0° for both. The nominal doping concentrations were approximately mid-10^18^ cm^–3^ for both. Sample ID: [MU786].

Results for Zn:AlGaAs are shown in Figure 7. Gradual smoothing is seen for the Zn:AlGaAs/B spall (Figure 7a). The smoothing for Zn:AlGaAs/A spall (Figure 7b) is more rapid but with the complication of voids. In this and other images (not shown), the void size decreases as the valley depth decreases, so a surface with less surface relief might prevent void formation altogether. Therefore, for both the A- and B-spalled substrates, Zn:AlGaAs looks like a promising smoothing material, in particular if the starting peak-to-valley height were a little smaller than it was for the samples used here.

### Zn:GaAs

**Figure 8:**
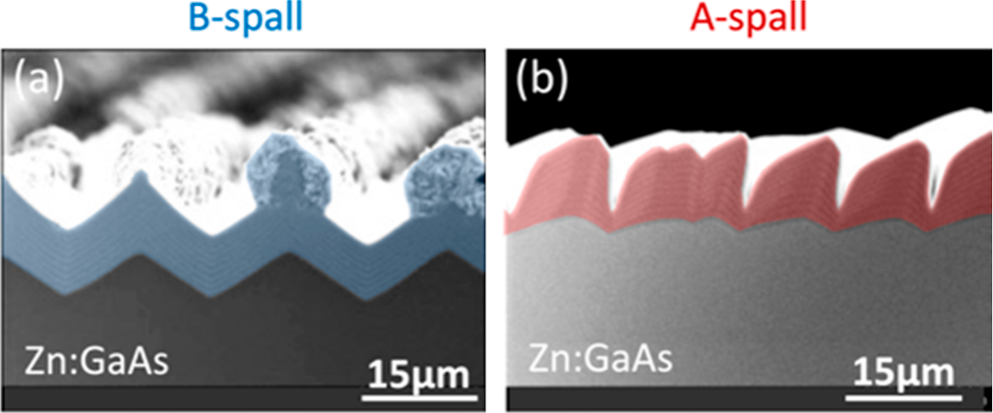
XSEM images of Zn:GaAs epilayer growth on spalled GaAs(100) substrates. Substrate offcuts: 6°A and 6°B for (a,b), respectively. The nominal doping concentrations were approximately mid-10^18^ cm^–3^ for both. Sample IDs: [MU388, 448].

A quite different behavior is seen for Zn:GaAs in Figure 8a, where no smoothing is observed. Instead, “mushroom” growth on the ridgetops is observed on a B-spalled substrate (Figure 8a), and deep trenches form on an A-spalled substrate (Figure 8b). While not useful for smoothing, this is still an informative result, because Zn:GaAs layers are often used in subsequent cell growth. This result indicates that some degree of smoothness is needed prior to Zn:GaAs growth to avoid the roughening seen here.

It should be mentioned that Zn:GaAs can be used effectively for substrate smoothing during hydride vapor-phase epitaxy (HVPE) growth.^17^ This difference is presumably linked to the differing growth environments for OMVPE and HVPE. One obvious difference is the presence of Cl in an HVPE environment, which may enable some smoothing processes not accessible to OMVPE (except perhaps when supplied by a source like CCl_4_). A more detailed study of substrate smoothing for HVPE growth has been conducted in a related study by Braun et al.^17^

### GaInP

**Figure 9:**
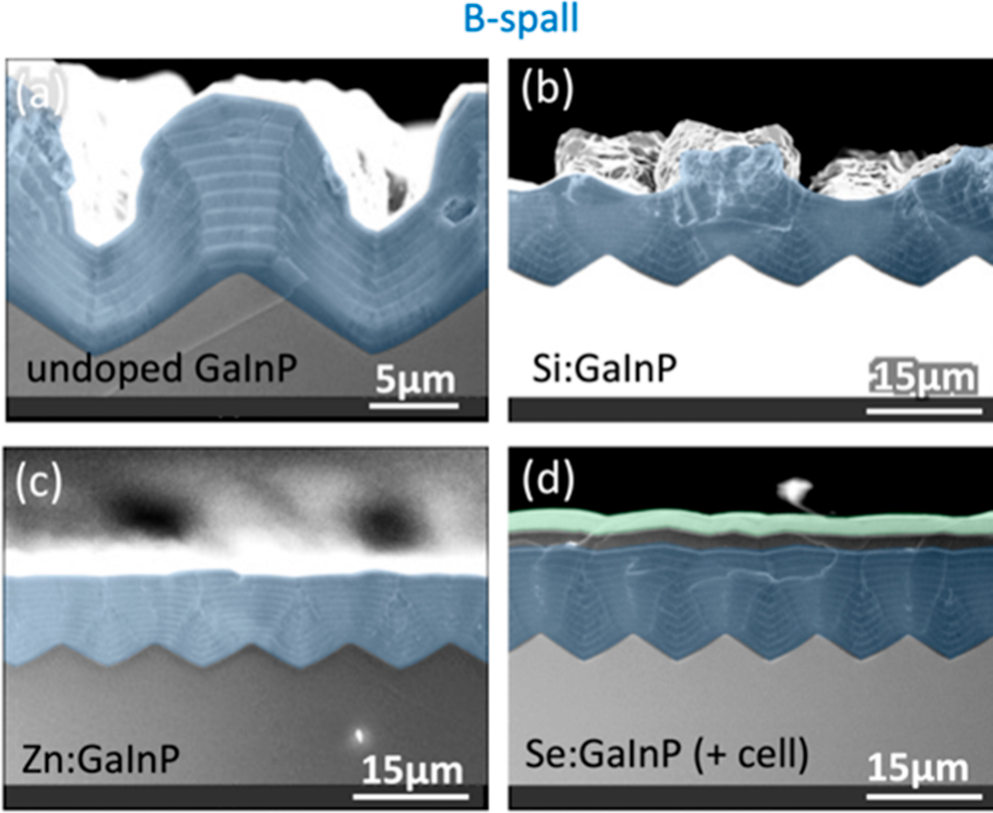
XSEM images for GaInP epilayers grown on B-spalled GaAs(100) substrates with (a) no doping and (b–d) Si, Zn, and Se doping, respectively. A lower-resolution image of (a) is shown in Figure 5c. Substrate offcuts: 6°A, 0°, 6°A, and 6°A for (a–d), respectively. The nominal doping concentrations for (b–d) were approximately mid-10^17^ cm^–3^. Sample IDs: [MU382, 784, 384, 797].

In Figure 9, GaInP was grown on B-spalled substrates with different dopants. When GaInP is undoped (Figure 9a), it grows faster atop the ridges, roughening the sample. In addition, some evidence for stabilization of nominally {011} sidewall facets between the ridgetops and valleys can also be seen. Some smoothing is seen for Si:GaInP (Figure 9b), but there are also many ridgetop “mushrooms” which roughen the surface.

Gradual smoothing is seen for Zn:GaInP and Se:GaInP (Figure 9c,d). In both cases, this smoothing appears to be the result of nearly isotropic growth. In Figure 9d, solar cell device layers have been grown atop the smoothing layers. The two thickest layers in this sequence are a 2 μm Se:GaInP layer (shaded gray) and a 1.85 μm Zn:GaAs layer (shaded green); both appear to have grown conformally. Within the context of this paper, it is interesting to note that the Zn:GaAs layer does not exhibit the roughening seen in Figure 8, suggesting that the level of smoothing attained in Figure 9c might be sufficient for solar cell growth. The properties of a fully processed cell grown on a spalled substrate using a Se:GaInP smoothing layer have been measured as part of a related study,^6^ and the cell quality was comparable to benchmark cells grown on planar epiready GaAs(100).

### Combinations of Materials

**Figure 10:**
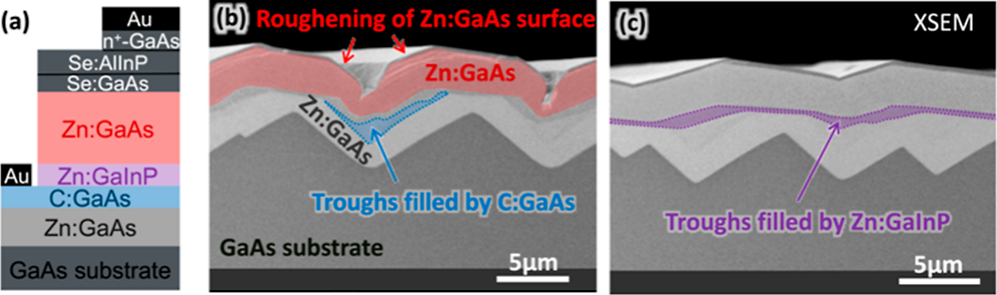
XSEM images illustrating some of the complications that occur if cells are grown on a faceted substrate without first growing a smoothing layer. These images also illustrate how some of the morphology changes seen in isolation elsewhere in this study can also be observed during more complex growth sequences. (a) Intended layer sequence for a GaAs cell, with relevant layers highlighted with color. (Further cell details can be found in ref (6).) In (b), the C:GaAs is smoothing the surface and Zn:GaAs is becoming rougher. (c) Some smoothing also occurs for the Zn:GaInP. This substrate is (acoustically) A-spalled GaAs(100) offcut 6° toward (111)A. The growth conditions for the four labeled layers are as follows: lower Zn:GaAs {700 °C, 6 μm/h, V/III = 250, TMGa}, C:GaAs {650 °C, 3 μm/h, V/III = 12, TMGa}, Zn:GaInP {650 °C, 4.4 μm/h, V/III = 86, TMGa}, upper Zn:GaAs {650 °C, 6.7 μm/h, V/III = 200, TEGa}, with all other gas precursors as listed in the “Experimental Details” section of the text. The nominal doping concentrations for the labeled layers were approximately low-10^18^ cm^–3^ (lower Zn:GaAs), > 10^19^ cm^–3^ (C:GaAs), 10^18^ cm^–3^ (Zn:GaInP), and 10^17^ cm^–3^ (upper Zn:GaAs). Sample ID: [MU116].

Although the principal motivation for this study was to identify OMVPE-grown smoothing layers for faceted GaAs(100), a broad understanding of the various growth characteristics of different dopant/material combinations can be very useful for understanding and debugging more complex situations. To provide an example of this, Figure 10 includes two XSEM images of an attempt to grow the layers needed for a GaAs solar cell on a spalled substrate without smoothing layers, using the sequence of layers shown in Figure 10a. Figures 10b,c shows the same layers grown on a spalled substrate (with no contact metallization or any other solar cell processing steps).

One thing these images demonstrate is the need for substrate smoothing prior to solar cell growth, because discontinuous layers and deep trenches typically hinder the processing and function of a solar cell. In the context of this study, though, they also show that some of the morphological changes seen in isolation elsewhere in this article can be observed in the midst of a more complex stack of materials.

The C:GaAs layer highlighted in Figure 10b has started to fill in a valley but not completely. As was also seen for the earlier layers in Figure 6c, very little C:GaAs has grown atop the adjacent ridges. This might not be a problem for substrate smoothing, but it could become a problem if a contiguous layer of uniform thickness were needed as part of a device structure. The Zn:GaInP layer highlighted in Figure 10c also fills in valleys but in the more conformal way seen in Figure 9c. This layer is contiguous and more uniformly thick; perhaps it could be used as part of a device structure, but this will be situational.

The Zn:GaAs layer highlighted in Figure 10b displays the roughening behavior seen in Figure 8. This would typically be undesirable for most device layers and would also likely create problems for subsequently grown layers. One possible consequence of Zn:GaAs-induced roughening is that subsequently grown thin layers may not be uniformly thick and/or might have pinholes (which could be particularly problematic for a passivating layer like a window layer). The amount of roughness that any given dopant/material combination can tolerate is highly situational and would likely need to be determined on a case-by-case basis. In general, though, some substrate smoothing will be needed prior to the growth of Zn:GaAs (by OMVPE) or other dopant/material combinations which exacerbate existing surface roughness.

### Voids and Supersteps

**Figure 11:**
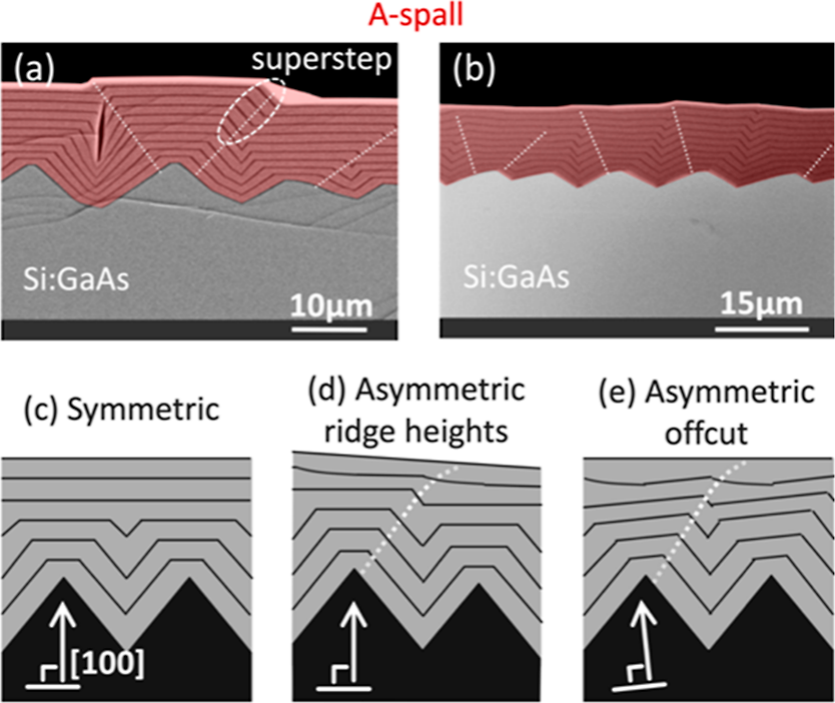
(a,b) are XSEM images of Si:GaAs grown on spalled GaAs(100) The voids in (a) occur only when the valley depth exceeds ∼5 μm. The Si doping in (a) is 100× greater than in (b), suggesting that the more persistent supersteps in (a) may be related to stabilization of faceted superstep faces by Si. The superstep encircled in (a) is created by filling of an asymmetric valley. Examples of this are indicated with dashed lines in (a,b). The creation of supersteps is related to substrate asymmetry: (c) Growth on a symmetric substrate without supersteps. (d,e) Dashed lines following superstep formation on asymmetric samples. In (d), the asymmetry is caused by differing ridge heights. The asymmetry in (e) is caused by an offcut with a downhill direction to the right. [Over the small region shown, (d,e) are crystallographically identical.] Substrate offcuts: 0° and 6°B for (a,b), respectively. The nominal doping concentrations for (a,b) were approximately mid-10^18^ and high-10^17^ cm^–3^, respectively. Sample IDs: [MU729, 454].

Two common complications that can occur during the growth of smoothing layers on spalled substrates are voids (discussed above) and supersteps (Figure 11).

Voids and deep trenches have been seen in several of the examples in this paper and are always located above valleys on the substrate surface. Because voids typically do not form in epilayers grown on planar epiready commercial substrates, there logically must be some roughness threshold below which voids do not form, and evidence for this was found for the materials used in this study.

In Figure 11a, there is a void above the deepest valley, but the adjacent shallower valleys do not support void formation. Figure 11b is also void-free and has shallower valleys. Although we have not conducted a systematic study of void formation versus valley depth, we have seen many samples with voids or deep trenches in some regions but not others, and their formation appears to correlate with ridge height. Based upon our preliminary results, it appears that the formation of voids and trenches becomes more prevalent once the ridge heights exceed ∼5 μm.

Localized offcut variations, supersteps, and localized faceting are all common surface features seen for epilayers grown on spalled substrates. To facilitate discussion, some definition for how these terms will be used here is useful: When individual surface steps are far apart, but the surface is nonplanar, the variations in surface orientation (and thereby step spacing) will be described as a variation in the local offcut angle. When these steps are bunched together with little or no space between them, they become a superstep. As growth proceeds, the individual steps in a superstep ideally move away from one another (dissipate) to flatten the surface. If instead a superstep structurally transforms into a localized facet (with a lower energy than the superstep configuration), it will tend to persist as a stable surface-roughening feature during subsequent growth.

Figure 11c–e illustrates different scenarios for superstep formation and dissipation during growth on a faceted substrate. Figure 11c is a symmetric case in which smoothing can occur with no superstep formation. Figure 11d and Figure 11e both have an asymmetry which creates supersteps. Figure 11d illustrates a case in which ridges of different heights on a nonoffcut (100) substrate create an asymmetric valley. Dashed lines show how the filling of this asymmetric valley creates a superstep in the fourth marker layer. In this example, subsequent growth broadens this superstep to create a planar surface (with regularly spaced individual surface steps), as indicated by the fifth and sixth marker layers. Figure 11e mimics the sequence of events shown in Figure 11d but on an intentionally offcut substrate.

Some superstep examples have been marked with dashed lines in Figures 11a,b. In Figure 11a, these supersteps seem to become persistent facets that do not dissipate, and this might become a problem for subsequently grown layers. In Figure 11b, the Si doping concentration is 100 times smaller, and some supersteps do dissipate, creating a more planar surface. This suggests that surface impurity/dopant concentrations can affect whether or not a superstep transforms into a stable facet.

The results presented above suggest some measures that can be taken to minimize the formation and persistence of supersteps. The first is related to the sample offcut direction. If an offcut is needed, an uphill–downhill direction orthogonal to the spall direction will avoid the asymmetry seen in Figure 11e. A planar spall is also helpful to avoid local offcut variations due to variable ridge heights.

Finally, the persistence or dissipation of any remaining supersteps will depend on the dopant/material combination being used. The Zn:GaAs in Figure 8 would likely be a poor choice (for OMVPE) because it tends to create persistent or steepening facets. In contrast, the C:GaAs/A spall in Figure 6d contains several supersteps which dissipate. Se:GaInP and several other materials also seem to dissipate supersteps. The number of different combinations for dopant, material, and growth conditions is enormous, so the examples shown here represent just a few possible solutions.

### Discussion of Surface Effects

In principle, it might be possible to predict the morphological evolution that would be seen for any given combination of starting morphology, material, surface impurity/dopant, and growth conditions. However, one of the inputs for such a theoretical understanding would be the atomic structures of these surfaces, and these surface structures are complex and largely unknown. In addition, a comprehensive model would have to include the impact of steps and other deviations from perfectly faceted surfaces, and the atomic structures of these are also largely unknown. Fortunately, an understanding of some basic principles is enough to inform the experimental design underlying empirical experimental studies.

Studies of growth on patterned substrates using molecular beam epitaxy (MBE) have provided a basic understanding of how surface diffusion of adatoms from one facet to another can change their relative growth rates.^18,19^ These studies were able to focus on surface diffusion by choosing growth conditions for which the sticking coefficient is near unity with no adatoms leaving the surface. For OMVPE and HVPE, surface reactions can remove previously deposited adatoms from the surface, changing the sticking coefficient (and thereby the growth rates) on various facets and giving them different growth rates even in the absence of surface diffusion.^20^ Individually, changing either the surface diffusion of adatoms or their sticking coefficients can create the various morphological changes described in this paper. Combining the two effects creates more opportunities (and more complexity). In both cases, the growth conditions and spall direction can affect the resulting morphological evolution, by changing both the sticking coefficient and surface mobility of adatoms on the surface. (The morphological evolution of epilayers grown on patterned substrates is analogous to the work presented here and can provide some useful insights.^21−28^).

An additional factor is that incorporation of dopant/impurity atoms at a surface can dramatically alter its structure and characteristics. Semiconductor surfaces generally reconstruct to reduce the number of partially filled (“dangling”) bonds, and impurity (dopant) atoms typically find bonding sites which further alter the surface structure.^29,30^ This can affect both the mobility of adatoms diffusing across the surface and the sticking coefficient of adatoms impinging on it, which in turn alters the morphological evolution of the surface. There have been many MBE studies of surface reconstructions containing surface impurity/dopant atoms (e.g., refs (31−33)) and of impurities/dopants or surfactants affecting surface morphology during growth (e.g., refs (12,28)), but much less is known about the atomic structure of surfaces in an OMVPE environment.

The current study was partially motivated by some of our prior work in which some surface impurities and/or dopants dramatically altered the structure of the OMVPE-prepared surfaces. As observed by scanning tunneling microscopy (STM) and low-energy electron diffraction, some OMVPE-prepared III–V(100) surfaces intentionally exposed to adatoms from the set {C, Si, Ge, In, As} exhibit a nearly flawless “3 × 1” reconstruction.^34^ Furthermore, these 3 × 1 surfaces remained clean and easy to image with STM for days, suggesting that they are very low-energy surfaces with low sticking coefficients and (probably) high surface adatom mobilities. Although we never established the atomic structure for our observed 3 × 1 reconstructions, they are likely related to other reported 3 × 1 and 3 × 2 reconstructions created by delta doping of III–V(100) surfaces.^35−38^

Some prior studies suggest that these ″3 × 1″ surfaces might affect the morphological evolution of a III–V surface grown in an OMVPE environment. During GaP growth on offcut Si(100), there is evidence that Si from the substrate can create stabilized (100) facets, thereby altering the surface morphology.^15^ Both explicit and background Si doping of GaP surfaces has also been used to flatten GaP surfaces during GaP growth on v-grooved Si(100).^26^

Finally, different results can be expected for MBE, OMVPE, and HVPE, because each growth method can supply additional elements to the surface through various pathways. The reconstruction of Ga_*x*_In_1–*x*_P(100) is qualitatively different in OMVPE than in MBE, because OMVPE-prepared surfaces incorporate atomic hydrogen provided by precursors like PH_3_^39,40^ and AsH_3_.^41^ For HVPE, the availability of Cl to the surface similarly creates possibilities not available to OMVPE or MBE.^17^ Etching pathways unique to OMVPE and HVPE are also enabled by reactive species like atomic H and Cl, which can react with surface atoms to form stable molecules that are then carried away from the surface in the vapor phase.

## Conclusions

Spalling offers the promise of a low-cost method for removing solar cells from substrates for substrate reuse. However, the surfaces of GaAs(100) substrates can be faceted after spalling, so a method for smoothing them prior to subsequent solar cell growth is desired.

This work investigated substrate smoothing during OMVPE epilayer growth and found that the results depend strongly upon the materials, surface impurities/dopants, and the sample/spall orientation. These dependencies are most logically linked to how changes in surface structure and chemistry affect the surface diffusion of adatoms and their preferential attachment at different surface sites.

The results provide guidance for developing epilayer sequences for growing solar cells on faceted surfaces using OMVPE. For example, C:GaAs and Zn:GaInP preferentially fill in valleys and can therefore be used for substrate smoothing. In contrast, Zn:GaAs can exaggerate the existing roughness, so the surface should be smoothed prior to Zn:GaAs growth. Finally, it should be noted that the results here are for OMVPE growth. HVPE offers some additional promising results related to its differing growth environment, as evidenced by the ability to use Zn:GaAs as a smoothing layer.^5^ The differing results for these different growth techniques offer opportunities for further understanding.

## References

[ref1] Horowitz K.; Ptak A.; Smith B.; Remo T. A Techno-Economic Analysis and Cost Reduction Roadmap for III-V Solar Cells NREL/TP-6A20–72103, 2018.

[ref2] Ward J. S.; Remo T.; Horowitz K.; Woodhouse M.; Sopori B.; VanSant K.; Basore P. Techno-economic analysis of three different substrate removal and reuse strategies for III-V solar cells. Prog. Photovoltaics 2016, 24, 1284–1292. 10.1002/pip.2776.

[ref3] Arakawa K.; Takahashi K. Relationships between fracture parameters and fracture surface roughness of brittle polymers. Int. J. Fract. 1991, 48, 103–114. 10.1007/BF00018393.

[ref4] Bedell S. W.; Shahrjerdi D.; Hekmatshoar B.; Fogel K.; Lauro P. A.; Ott J. A.; Sosa N.; Sadana D. Kerf-Less Removal of Si, Ge, and III-V Layers by Controlled Spalling to Enable Low-Cost PV Technologies. IEEE J. Photovolt. 2012, 2, 141–147. 10.1109/JPHOTOV.2012.2184267.

[ref5] Braun A. K.; Theingi S.; McMahon W. E.; Ptak A. J.; Packard C. E. Controlled spalling of (100)-oriented GaAs with a nanoimprint lithography interlayer for thin-film layer transfer without facet formation. Thin Solid Films 2022, 742, 139049 10.1016/j.tsf.2021.139049.

[ref6] Schulte K. L.; Johnston S. W.; Braun A. K.; Boyer J. T.; Neumann A. N.; McMahon W. E.; Young M.; Coll P. G.; Bertoni M. I.; Warren E. L.; et al. GaAs solar cells grown on acoustically spalled GaAs substrates with 27% efficiency. Joule 2023, 7, 1529–1542. 10.1016/j.joule.2023.05.019.

[ref7] Chen J.; Packard C. E. Controlled spalling-based mechanical substrate exfoliation for III-V solar cells: A review. Sol. Energy Mater. Sol. Cells 2021, 225, 111018 10.1016/j.solmat.2021.111018.

[ref8] Coll P. G.; Neumann A.; Smith D.; Warren E.; Polly S.; Hubbard S.; Sonic Lift-off of GaAs-based Solar Cells with Reduced Surface Facets. In 2021 IEEE 48th Photovoltaic Specialists Conference (PVSC), 2021; pp 2141–2143.

[ref9] Neumann A. N.; Coll P. G.; Bertoni M. I.; Steiner M. A.; Warren E. L. Wet-Etching of Acoustically Spalled GaAs for Substrate Reuse. IEEE J. Photovolt. 2024, 14, 281–287. 10.1109/JPHOTOV.2024.3355405.

[ref10] Schwoebel R. L.; Shipsey E. J. Step Motion on Crystal Surfaces. J. Appl. Phys. 1966, 37, 3682–3686. 10.1063/1.1707904.

[ref11] Jeong H.-C.; Williams E. D. Steps on surfaces: experiment and theory. Surf. Sci. Rep. 1999, 34, 171–294. 10.1016/S0167-5729(98)00010-7.

[ref12] Wixom R. R.; Rieth L. W.; Stringfellow G. B. Te surfactant effects on the morphology of patterned (001) GaAs homoepitaxy. J. Cryst. Growth 2004, 269, 276–283. 10.1016/j.jcrysgro.2004.05.077.

[ref13] Biasiol G.; Kapon E. Mechanisms of Self-Ordering of Quantum Nanostructures Grown on Nonplanar Surfaces. Phys. Rev. Lett. 1998, 81, 2962–2965. 10.1103/PhysRevLett.81.2962.

[ref14] Biasiol G.; Gustafsson A.; Leifer K.; Kapon E. Mechanisms of self-ordering in nonplanar epitaxy of semiconductor nanostructures. Phys. Rev. B 2002, 65, 205306 10.1103/PhysRevB.65.205306.

[ref15] McMahon W. E.; Warren E. L.; Kibbler A. E.; France R. M.; Norman A. G.; Reedy R. C.; Olson J. M.; Tamboli A. C.; Stradins P. Surfaces and interfaces governing the OMVPE growth of APD-free GaP on AsH_3_-cleaned vicinal Si(100). J. Cryst. Growth 2016, 452, 235–239. 10.1016/j.jcrysgro.2016.05.014.

[ref16] Kim Y.; Park Y. K.; Kim M.-S.; Kang J.-M.; Kim S.-I.; Hwang S.-M.; Min S. K. Facet evolution of CCl4-doped multilayers during metalorganic chemical vapor deposition on patterned GaAs substrates. J. Cryst. Growth 1995, 156, 169–176. 10.1016/0022-0248(95)00210-3.

[ref17] Braun A. K.; Boyer J. T.; Schulte K. L.; McMahon W. E.; Simon J.; Perna A. N.; Packard C. E.; Ptak A. J. 24% Single Junction GaAs Solar Cell Grown Directly on Growth-Planarized Facets using Hydride Vapor Phase Epitaxy. Adv. Energy Mater. 2024, 14, 2302035 10.1002/aenm.202302035.

[ref18] Nilsson S.; Van Gieson E.; Arent D. J.; Meier H. P.; Walter W.; Forster T. Ga adatom migration over a nonplanar substrate during molecular beam epitaxial growth of GaAs/AlGaAs heterostructures. Appl. Phys. Lett. 1989, 55, 972–974. 10.1063/1.101693.

[ref19] Koshiba S.; Nakamura Y.; Noda T.; Watanabe S.; Akiyama H.; Sakaki H. Transformation of GaAs (001)-(111)B facet structure by surface diffusion during molecular beam epitaxy on patterned substrates. J. Cryst. Growth 2001, 227–228, 62–66. 10.1016/S0022-0248(01)00633-9.

[ref20] Shaw D. W. Kinetic aspects in the vapour phase epitaxy of III-V compounds. J. Cryst. Growth 1975, 31, 130–141. 10.1016/0022-0248(75)90122-0.

[ref21] Metaferia W.; Kataria H.; Sun Y.-T.; Lourdudoss S. Growth of InP directly on Si by corrugated epitaxial lateral overgrowth. J. Phys. D: Appl. Phys. 2015, 48, 045102 10.1088/0022-3727/48/4/045102.

[ref22] Lee S. C.; Huffaker D. L.; Brueck S. R. J. Faceting of a quasi-two-dimensional GaAs crystal in nanoscale patterned growth. Appl. Phys. Lett. 2008, 92, 023103 10.1063/1.2830988.

[ref23] Mangum J. S.; Theingi S.; Steiner M. A.; McMahon W. E.; Warren E. L. Development of High-Efficiency GaAs Solar Cells Grown on Nanopatterned GaAs Substrates. Cryst. Growth Des. 2021, 21, 5955–5960. 10.1021/acs.cgd.1c00835.

[ref24] Li Q.; Lau K. M. Epitaxial growth of highly mismatched III-V materials on (001) silicon for electronics and optoelectronics. Prog. Cryst. Growth Charact. Mater. 2017, 63, 105–120. 10.1016/j.pcrysgrow.2017.10.001.

[ref25] Li J. Z.; Bai J.; Major C.; Carroll M.; Lochtefeld A.; Shellenbarger Z. Defect reduction of GaAs/Si epitaxy by aspect ratio trapping. J. Appl. Phys. 2008, 103, 106102 10.1063/1.2924410.

[ref26] Saenz T. E.; Mangum J. S.; Schneble O. D.; Neumann A. N.; France R. M.; McMahon W. E.; Zimmerman J. D.; Warren E. L. Coalescence of GaP on V-Groove Si Substrates. ACS Appl. Electron. Mater. 2023, 5, 721–728. 10.1021/acsaelm.2c01688.

[ref27] Kunert B.; Guo W.; Mols Y.; Tian B.; Wang Z.; Shi Y.; Van Thourhout D.; Pantouvaki M.; Van Campenhout J.; Langer R.; et al. III/V nano ridge structures for optical applications on patterned 300 mm silicon substrate. Appl. Phys. Lett. 2016, 109, 091101 10.1063/1.4961936.

[ref28] Bae S.-Y.; Lekhal K.; Lee H.-J.; Min J.-W.; Lee D.-S.; Honda Y.; Amano H. Selective-area growth of doped GaN nanorods by pulsed-mode MOCVD: Effect of Si and Mg dopants. Phys. Status Solidi 2017, 254, 1600722 10.1002/pssb.201600722.

[ref29] Duke C. B. Semiconductor Surface Reconstruction: The Structural Chemistry of Two-Dimensional Surface Compounds. Chem. Rev. 1996, 96, 1237–1260. 10.1021/cr950212s. 11848788

[ref30] Xue Q.-K.; Hashizume T.; Sakurai T. Scanning tunneling microscopy of III-V compound semiconductor (001) surfaces. Prog. Surf. Sci. 1997, 56, 1–131. 10.1016/S0079-6816(97)00033-6.

[ref31] Ohtake A.; Hanada T.; Yasuda T.; Yao T. Adsorption of Zn on the GaAs(001)-(2×4) surface. Appl. Phys. Lett. 1999, 74, 2975–2977. 10.1063/1.123984.

[ref32] Miotto R.; Srivastava G. P.; Ferraz A. C. Theoretical studies of the initial stages of Zn adsorption on GaAs(001)-(2 × 4). Phys. Rev. B 2000, 62, 13623 10.1103/PhysRevB.62.13623.

[ref33] Dadras J.; Park J. H.; Ratsch C. Effects of dopants on electronic surface states in InAs. Phys. Rev. B 2019, 99, 245406 10.1103/PhysRevB.99.245406.

[ref34] McMahon W. E.; (unpublished).

[ref35] Li L.; Qi H.; Gan S.; Han B. K.; Hicks R. F. Site-specific chemistry of carbon tetrachloride decomposition on GaAs(001). Appl. Phys. A: Mater. Sci. Process. 1998, 66, S501–S505. 10.1007/s003390051191.

[ref36] Wassermeier M.; Behrend J.; Däweritz L.; Ploog K. Reconstruction of the GaAs(001) surface induced by submonolayer Si deposition. Phys. Rev. B 1995, 52, R2269–R2272. 10.1103/PhysRevB.52.R2269. 9981388

[ref37] Sauvage-Simkin M.; Garreau Y.; Pinchaux R.; Coati A.; Ouerghi A.; Etienne B. Atomic structure of the (3×2) Si–GaAs (001) reconstructed surface: A clue to δ doping mechanism derived from in situ grazing incidence X-ray diffraction data. Surf. Sci. 2010, 604, 415–419. 10.1016/j.susc.2009.12.004.

[ref38] Wassermeier M.; Kellermann S.; Behrend J.; Däweritz L.; Ploog K. Submonolayer Si deposition at low temperatures on the GaAs(001)-(2×4) surface studied by scanning tunneling microscopy. Surf. Sci. 1998, 414, 298–303. 10.1016/S0039-6028(98)00537-8.

[ref39] Chen G.; Cheng S. F.; Tobin D. J.; Li L.; Raghavachari K.; Hicks R. F. Indium phosphide (001)-(2×1): Direct evidence for a hydrogen-stabilized surface reconstruction. Phys. Rev. B 2003, 68, 121303 10.1103/PhysRevB.68.121303.

[ref40] Batyrev I. G.; McMahon W. E.; Zhang S. B.; Olson J. M.; Wei S.-H. Step Structures on III-V Phosphide (001) Surfaces: How Do Steps and Sb affect CuPt Ordering of GaInP_2_?. Phys. Rev. Lett. 2005, 94, 096101 10.1103/PhysRevLett.94.096101. 15783978

[ref41] Karmo M.; Ruiz Alvarado I. A.; Schmidt W. G.; Runge E. Reconstructions of the As-Terminated GaAs(001) Surface Exposed to Atomic Hydrogen. ACS Omega 2022, 7, 5064–5068. 10.1021/acsomega.1c06019. 35187322 PMC8851457

